# Supplementary material for: The impact of face masks on emotion recognition performance and perception of threat
Source: PLoS One. 2022 Feb 11;17(2):e0262840. doi: 10.1371/journal.pone.0262840 (PMC8836371; doi:10.1371/journal.pone.0262840)
Supplement: S1 File — (PDF) [file pone.0262840.s001.pdf]

The impact of face masks on emotion recognition performance and perception of threat

Melina Grahlow, Claudia Ines Rupp & Birgit Derntl

- Supporting Information -

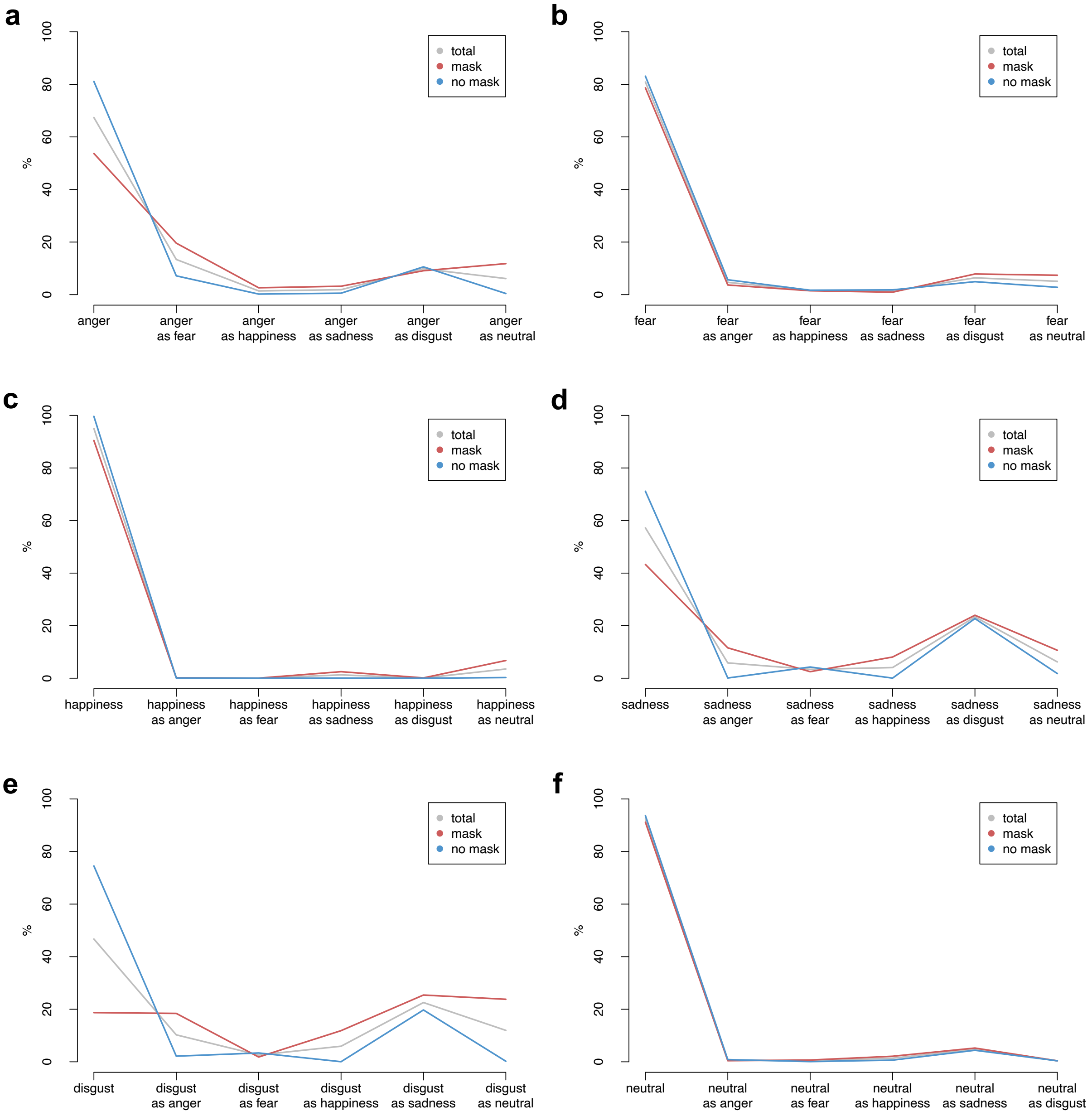

S1 Fig 1. Misinterpretation of emotions in the facial emotion recognition task. Mean responses in the emotion recognition task (in percent) for (a) anger, (b) fear, (c) happiness, (d) sadness, (e) disgust and (f) neutral with depiction of misinterpretations of one emotion as another emotion in total and for faces with and without face mask separately (n = 790).

S1 Table 1. Pseudo-randomised order of stimuli for the two versions A and B used in the experimental condition *mask vs. original*.

Version A					Version B			
Female		Male			Female		Male	
anger	young			1			anger	old
		anger	*old*	2	*anger*	*young*		
		happiness	old	3	neutral	medium		
happiness	*medium*			4			sadness	old
anger	old			5			*neutral*	*old*
		disgust	*medium*	6	disgust	medium		
		sadness	young	7			*anger*	*young*
fear	*young*			8	*anger*	*old*		
		anger	young	9			sadness	medium
		fear	*medium*	10	*fear*	*old*		
fear	medium			11	happiness	medium		
disgust	*young*			12			*fear*	*old*
		disgust	young	13	disgust	young		
		happiness	medium	14			*happiness*	*young*
happiness	*young*			15			fear	medium
neutral	young			16	happiness	young		
		fear	old	17	*sadness*	*medium*		
neutral	*medium*			18			*happiness*	*medium*
		sadness	*medium*	19	sadness	young		
sadness	old			20	*fear*	*medium*		
sadness	*young*			21			*happiness*	*old*
		neutral	*medium*	22			fear	young
sadness	medium			23	neutral	old		
		neutral	young	24			*sadness*	*young*
		fear	*young*	25	disgust	old		
happiness	old			26			*disgust*	*young*
disgust	*old*			27			disgust	medium
		anger	*medium*	28	*neutral*	*young*		
		happiness	young	29			disgust	old
disgust	*medium*			30	*happiness*	*old*		
anger	medium			31	fear	young		
		neutral	old	32			*neutral*	*young*
		disgust	*old*	33			neutral	medium
fear	old			34	*sadness*	*old*		
		sadness	*old*	35			anger	medium
neutral	*old*			36	*anger*	*medium*		

The 72 adapted stimuli used in the experimental condition *mask vs. original* were split into two versions of 36 stimuli so that each poser was only seen once in each version (if male poser 'anger & old' was wearing a mask in version A, the same stimulus was presented without a face mask in version B). Both versions were balanced for emotion, sex, age and face mask and the order of stimuli was drawn randomly. Stimuli with face masks are typeset in italics, original stimuli in roman type.

S1 Table 2. Emotion-specific results from regression analyses on the association of participant age with emotion recognition accuracy for faces with and without face mask.

Condition	Emotion	*F*	*p*	R^2	*β*
Mask	Anger	12.79	< .001*	.02	-.003
	Fear	9.24	.002*	.01	-.002
	Happiness	1.36	.244		
	Sadness	0.30	.585		
	Disgust	13.75	< .001*	.02	-.002
	Neutral	12.20	.001*	.02	-.002
Original	Anger	0.60	.438		
	Fear	3.37	.067		
	Happiness	0.03	.865		
	Sadness	8.15	.004*	.01	.002
	Disgust	4.78	.029*	.01	-.002
	Neutral	15.62	< .001*	.02	-.002

Note. Results from regression analyses on the association of participant age with emotion recognition accuracy (percent correct) for faces with ('mask') and without face masks ('original') specific for each emotion (n = 790). For significant results, R^2 *and the β-coefficient indicating the direction of the correlation are provided.*
** p < .05*

S1 Table 3. Differences in emotion recognition accuracy (percent correct) between the different conditions.

Emotion	Condition		*M*	*SD*	*p*
Anger	original		0.85	0.22	
		vs. mask			< .001*
		vs. bubble			< .001*
		vs. half			< .001*
	mask		0.56	0.31	
		vs. bubble			.069
		vs. half			.618
	bubble		0.59	0.16	
		vs. half			1.000
	half		0.58	0.18	
Fear	original		0.83	0.24	
		vs. mask			.001*
		vs. bubble			< .001*
		vs. half			< .001*
	mask		0.79	0.24	
		vs. bubble			.182
		vs. half			< .001*
	bubble		0.76	0.21	
		vs. half			.372
	half		0.73	0.21	
Happiness	original		0.99	0.03	
		vs. mask			< .001*
		vs. bubble			< .001*
		vs. half			< .001*
	mask		0.92	0.17	
		vs. bubble			.001*
		vs. half			< .001*
	bubble		0.89	0.13	
		vs. half			1.000
	half		0.88	0.14	
Sadness	original		0.71	0.29	
		vs. mask			< .001*
		vs. bubble			< .001*
		vs. half			< .001*
	mask		0.43	0.30	
		vs. bubble			.231
		vs. half			1.000
	bubble		0.47	0.21	
		vs. half			.204
	half		0.43	0.22	
Disgust	original		0.80	0.28	
		vs. mask			< .001*
		vs. bubble			< .001*
		vs. half			< .001*
	mask		0.11	0.18	
		vs. bubble			< .001*
		vs. half			< .001*
	bubble		0.26	0.17	
		vs. half			.057
	half		0.22	0.15	
Neutral	original		0.94	0.15	
		vs. mask			.016*
		vs. bubble			.001*
		vs. half			< .001*
	mask		0.91	0.17	
		vs. bubble			1.000
		vs. half			.001*
	bubble		0.90	0.14	
		vs. half			.174
	half		0.87	0.19	

Significant effects obtained through analysis of variance were followed up with Bonferroni-corrected multiple comparisons to analyse differences in emotion recognition accuracy (percent correct) between the different conditions for each emotion separately. *Mask vs. original* (n = 790), *half vs. bubble* (n = 395 and n = 388).
* $p < .05$

S1 Table 4. Differences in rating of threat between faces with and without a mask for correctly recognised stimuli only.

Emotion	Condition	*M*	*SD*	*p*
Anger	mask	73.88	16.69	.002 *
	original	76.93	17.45	
Fear	mask	38.88	23.33	1.000
	original	38.30	23.18	
Happiness	mask	12.38	13.43	< .001 *
	original	9.49	12.10	
Sadness	mask	30.47	21.82	< .001 *
	original	23.15	18.45	
Disgust	mask	40.44	22.37	< .001 *
	original	32.56	22.49	
Neutral	mask	23.22	18.55	.460
	original	21.70	17.15	

Significant effects obtained through analysis of variance were followed up with Bonferroni-corrected multiple comparisons to analyse differences in rating of threat on a Visual Analogue Scale with a range from 0 to 100 between the different conditions for each emotion separately for correctly recognised 'mask' and 'original' stimuli only (n = 790).
* $p < .05$

S1 Table 5. Emotion-specific results from regression analyses on the association of participant age with rating of threat for faces with and without face mask.

Condition	Emotion	*F*	*p*	R^2	*β*
Mask	Anger	23.71	< .001*	.03	-.259
	Fear	2.08	.149		
	Happiness	0.02	.879		
	Sadness	5.73	.017*	.01	-.132
	Disgust	8.00	.005*	.01	-.231
	Neutral	3.57	.059		
Original	Anger	4.21	.041*	.01	-.106
	Fear	0.01	.935		
	Happiness	0.00	.971		
	Sadness	1.92	.166		
	Disgust	0.09	.761		
	Neutral	6.20	.013*	.01	-.118

Note. Results from regression analyses on the association of participant age with rating of threat on a Visual Analogue Scale with a range from 0 to 100 for faces with ('mask') and without face masks ('original') specific for each emotion (n = 790). For significant results, R^2 *and the β-coefficient indicating the direction of the correlation are provided.*
* $p < .05$

S1 Table 6. Differences in rating of threat between the different conditions.

Emotion	Condition		*M*	*SD*	*p*
Anger	original		74.24	18.44	
		vs. mask			< .001*
		vs. bubble			< .001*
		vs. half			< .001*
	mask		61.88	19.19	
		vs. bubble			.118
		vs. half			.894
	bubble		59.35	14.82	
		vs. half			1.000
	half		60.33	14.25	
Fear	original		39.94	22.71	
		vs. mask			1.000
		vs. bubble			.823
		vs. half			1.000
	mask		39.49	21.76	
		vs. bubble			1.000
		vs. half			.540
	bubble		37.95	20.94	
		vs. half			.084
	half		41.75	19.34	
Happiness	original		9.55	12.21	
		vs. mask			< .001*
		vs. bubble			.008*
		vs. half			< .001*
	mask		13.30	13.76	
		vs. bubble			.533
		vs. half			.736
	bubble		12.00	10.87	
		vs. half			.030*
	half		14.48	10.90	
Sadness	original		25.26	17.74	
		vs. mask			< .001*
		vs. bubble			.070
		vs. half			< .001*
	mask		34.35	19.67	
		vs. bubble			< .001*
		vs. half			1.000
	bubble		28.08	16.01	
		vs. half			< .001*
	half		33.81	17.09	
Disgust	original		32.59	21.68	
		vs. mask			< .001*
		vs. bubble			.654
		vs. half			1.000
	mask		49.10	29.28	
		vs. bubble			< .001*
		vs. half			< .001*
	bubble		30.34	14.37	
		vs. half			.191
	half		33.82	14.46	
Neutral	original		22.09	16.97	
		vs. mask			.936
		vs. bubble			.018*
		vs. half			.895
	mask		23.28	17.97	
		vs. bubble			< .001*
		vs. half			1.000
	bubble		19.02	13.69	
		vs. half			.001*
	half		23.57	15.72	

Significant effects obtained through analysis of variance were followed up with Bonferroni-corrected multiple comparisons to analyse differences in rating of threat on a Visual Analogue Scale with a range from 0 to 100 between the different conditions for each emotion separately. *Mask vs. original* (n = 790), *half vs. bubble* (n = 395 and n = 388).
* $p < .05$
