# Supplementary material for: The impact of face masks on emotion recognition performance and perception of threat
Source: PLoS One. 2022 Feb 11;17(2):e0262840. doi: 10.1371/journal.pone.0262840 (PMC8836371; doi:10.1371/journal.pone.0262840)
Supplement: Attachment — Submitted filename: Response_to_Reviewers.pdf [file pone.0262840.s003.pdf]

We would like to thank the reviewer and the Academic Editor for their constructive and insightful comments that helped improving our manuscript once more. Please find our response in detail below. Any changes within the manuscript are highlighted in yellow.

Academic Editor

Thank you for submitting your manuscript to PLOS ONE. After careful consideration, we feel that it has merit but does not fully meet PLOS ONE's publication criteria as it currently stands. Therefore, we invite you to submit a revised version of the manuscript that addresses the points raised during the review process.

Reviewer #2 and I have read the revision of your manuscript and your responses to the reviews. In our opinion, the quality of your work is substantially improved, and you were responsive to most of the comments. However, there are some points left which should be addressed in a further revision. Some restructuring of the manuscript is needed (see for details the attached comments of reviewer #2). The abstract should be updated, and age should already be mentioned in the introduction section as an important factor influencing facial emotion recognition. Your conclusions need also some rewording. Please check the access to the raw data of your investigation.

Response: Thank you very much for the opportunity to revise our manuscript and to resubmit once more. We appreciate the acknowledgement of our work so far and have paid special attention to address the remaining points raised.

Journal Requirements

Please review your reference list to ensure that it is complete and correct. If you have cited papers that have been retracted, please include the rationale for doing so in the manuscript text or remove these references and replace them with relevant current references. Any changes to the reference list should be mentioned in the rebuttal letter that accompanies your revised manuscript. If you need to cite a retracted article, indicate the article's retracted status in the References list and also include a citation and full reference for the retraction notice.

Response: Thank you for this comment. We have reviewed our reference list and ensured that it is complete and correct to the best of our knowledge. None of the papers cited have been retracted.

Reviewer 2

The authors have responded to most of my comments very well. I have a few new comments, mostly driven by the changes that have been made to the manuscript.

1) The structure of the manuscript could use some work now the results are analysed in one ANOVA. I don't think it makes sense to present them as Experiment 1 and Experiment 2 in the methods but analyse the experiments together. I would recommend introducing them as different conditions that were between versus within participant and then cluster the results by hypothesis (as they currently are). It can be clear that they were carried out in a serial order, so the methods and results can still capture the intention with which they were run. The abstract should also be updated in this way.

Response: We thank the reviewer for this useful hint and for suggesting a more suitable structure for the manuscript. We have restructured the manuscript accordingly and now introduce the experimental conditions as two different conditions that were carried out in a serial order: *mask vs. original* (former Experiment 1) and *half vs. bubble* (former Experiment 2). Please see the methods section, page 8:

*"**The current study comprised two experimental conditions. Condition mask vs. original** (with stimuli 'mask' and 'original'; please see Fig 1) **was conducted** by 790 participants (636 women) between 18 and 89 years (M = 30.85, SD = 12.68). **Condition half vs. bubble** consisted of two parallel versions using the stimuli 'half' and 'bubble', see Fig 1. Here, 395 participants (289 women) between 18 and 70 years (M = 29.16, SD = 11.12) **saw 'half' faces** and 388 participants (298 women) between 18 and 65 years (M = 28.94, SD = 10.83) **were presented with 'bubble' stimuli**."*

We have also updated the abstract in this regard:

*"Facial emotion recognition is crucial for social interaction. However, in times of a global pandemic, where wearing a face mask covering mouth and nose is widely encouraged to prevent the spread of disease, successful emotion recognition may be challenging. In the current study, we investigated whether emotion recognition, assessed by a validated emotion recognition task, is impaired for faces wearing a mask compared to uncovered faces, in a sample of 790 participants between 18 and 89 years (**condition mask vs. original**). In two more samples of 395 and 388 participants between 18 and 70 years, we assessed emotion recognition performance for faces that are occluded by something other than a mask, i.e., a bubble as well as only showing the upper part of the faces (**condition half vs. bubble**). **Additionally, perception of threat for faces with and without occlusion was assessed. We found impaired emotion recognition for faces wearing a mask compared to faces without mask, for all emotions tested (anger, fear, happiness, sadness, disgust, neutral). Further, we observed that perception of threat was altered for faces wearing a mask.** Upon comparison of the different types of occlusion, we found that, for most emotions and especially for disgust, there seems to be an effect that can be ascribed to the face mask specifically, both for emotion recognition performance and perception of threat. Methodological constraints as well as the importance of wearing a mask despite temporarily compromised social interaction are discussed."*

2) Given that age now seems to be explored in the results, it would be helpful to have some context related to this in the introduction. Line 310: for the regression with age, were responses collapsed across emotions? It could be clearer.

Response: We thank the reviewer for pointing out the missing information in the introduction and the results section. We have added information on the influence of age on emotion recognition in the introduction, please see page 5:

"One factor that might influence emotion recognition is the participant's age. Research investigating age differences report inconsistent emotion-specific effects: while emotion recognition seems to be less accurate for fear [27,28], anger [27–29], and sadness [28–30] in older participants (between 65-80 years of age), improved recognition with older age (between 58-70 years) has been observed for happiness [30] and disgust [29]."

Further, we have clarified the description of the regression analysis with age in the results section. We present results on the association of age with recognition accuracy for masked and unmasked faces in general in the results section, while emotion-specific results can be found in S2 Table, please see page 14:

"To check whether there is an effect of participant age on emotion recognition accuracy for masked and unmasked original faces ***in general as well as for each emotion****, we performed linear regression analyses. Results from the analysis on the association of participant age with emotion recognition accuracy for masked faces revealed a significant effect of age, $F(1, 788) = 30.21$, $p < .001$, $R^2 = .04$, with lower recognition rates for faces with a mask for older participants. In contrast, the analysis on the association of participant age with recognition rates for unmasked original faces did not reveal a significant effect, $F(1, 788) = 1.38$, $p = .241$. See S2 Table for emotion-specific results."*

3) The conclusion seems to be mainly based on evidence that wasn't provided in the current study (i.e., discussing the possibility of face masks hindering emotion perception in different disorders). Line 580: In many cases it's not our choice whether to accept the consequences of face masks (they have been mandated in much of the world), therefore this final sentence needs rewording.

Response: Thank you for raising this point. We followed the suggestions reviewer #1 provided during the last review process to frame the research topic of emotion recognition of faces with and without mask in a broader context and to emphasise possibly detrimental effects of face masks on social interaction for specific groups of individuals. Due to economic validity we decided to forego assessing mental health in the current study, however, we decided – also stimulated by reviewer #1's comments – to still discuss our findings in the context of mental disorders but drastically shortened this part of the discussion, please see page 24:

"To conclude, we were able to show that wearing a face mask affects social interaction in terms of impaired emotion recognition accuracy and altered perception of threat. ***This might be especially relevant and possibly detrimental for individuals with certain mental disorders who show altered emotion recognition, for instance individuals with autism spectrum disorder, major depressive disorder or alcohol use disorder. For these persons, face masks may pose an additional obstacle during social interaction which is already impeded due to the nature of their mental disorder [48]. Future studies on the effect of face masks on emotion recognition and social interaction should therefore additionally focus on measures of social competence and assess whether psychopathology moderates the effects of face masks on emotion recognition***."

Thank you for pointing out that our concluding sentence is not up to date anymore considering the evolving pandemic situation. We have rephrased accordingly, please see page 25:

"However, ***despite the limitations*** *posed by mask wearing* ***and the proposed effect on social interaction****, there are many positive consequences of wearing face masks, such as touching one's face less often [49], which could in turn prevent the spread of infectious diseases."*

4) In addition, I attempted to access the raw data from the DOI provided, but was unable to, with an error saying to try again later. It may be a problem at my end but would be helpful if the authors could double check.

Response: We thank the reviewer for making us aware that our raw data were not accessible. We have double checked the access and found that we could access the raw data. Please note that it might be necessary to download the provided csv-files instead of viewing the data in the repository itself. Please find the relevant DOI necessary to access our data in the Open Science Framework here: https://doi.org/10.17605/OSF.IO/JY2ES.
